# *Rickettsia conorii* Subspecies *israelensis* in Captive Baboons

**DOI:** 10.3201/eid2904.221176

**Published:** 2023-04

**Authors:** Giovanni Sgroi, Roberta Iatta, Grazia Carelli, Annamaria Uva, Maria Alfonsa Cavalera, Piero Laricchiuta, Domenico Otranto

**Affiliations:** Experimental Zooprophylactic Institute of Southern Italy, Portici, Italy (G. Sgroi);; University of Bari Aldo Moro, Bari, Italy (G. Sgroi, R. Iatta, G. Carelli, A. Uva, M.A. Cavalera, D. Otranto);; Zoosafari Park Fasano, Brindisi, Italy (P. Laricchiuta);; Bu-Ali Sina University, Hamedan, Iran (D. Otranto)

**Keywords:** rickettsia, zoonoses, *Rickettsia conorii*, tick-borne infections, vector-borne infections, primates, Italy, bacteria

## Abstract

Hamadryas baboons (*Papio hamadryas*) may transmit zoonotic vector-borne pathogens to visitors and workers frequenting zoological parks. We molecularly screened 33 baboons for vector-borne pathogens. Three (9.1%) of 33 animals tested positive for *Rickettsia conorii* subspecies *israelensis*. Clinicians should be aware of potential health risks from spatial overlapping between baboons and humans.

*Papio hamadryas* baboons (order Primates, family Cercopithecidae) are frequently hosted in zoological gardens worldwide. The natural susceptibility of baboons to many zoonotic agents (*1*) may present a potential risk for transmission of emerging infectious diseases to humans. Nevertheless, few data are available on vector-borne pathogens of human concern that are hosted by baboons (e.g., *Rickettsia africae*, *Babesia microti*–like parasites, and *Anaplasma phagocytophilum*) (*1*). Data are likewise scarce on the role of *P. hamadryas* baboons in circulating arthropod vectors in zoological gardens and the resulting risk for transmitting vector-borne pathogens to persons frequenting such areas. We aimed to determine the occurrence of zoonotic vector-borne pathogens in a zoopark in the Apulia region of southern Italy and assess baboons’ potential roles as reservoirs of emerging pathogens. Our study was approved by the University of Bari Aldo Moro ethics committee (Prot. Uniba 176/19). 

During February–December 2020, we anesthetized baboons in the zoopark and housed them in cages for blood sampling. For each baboon, we recorded age, sex, weight, and body condition score (1–5); we obtained peripheral blood samples by cephalic vein puncture. To determine complete blood count and for molecular analysis, we collected 2 mL blood samples in Vacutainer K3-EDTA tubes. For biochemical analysis, we collected an additional 5 mL blood in Vacutainer clot activator serum tubes and centrifuged (15 min at 1,500 × *g* at room temperature), then delivered it to the University of Bari Department of Veterinary Medicine (Bari, Italy). We extracted DNA using QIAGEN QIAamp DNA Blood and Tissue kits (https://www.qiagen.com) and molecularly tested for vector-borne pathogens (Table) (*2*–*4*). We purified and sequenced amplicons in both directions using a Big Dye Terminator v3.1 Cycle Sequencing Kit in an Applied Biosystems 3130 Genetic Analyzer (ThermoFisher, https://www.thermofisher.com), then edited and analyzed them using Geneious version 9.0 (https://www.geneious.com). We then compared resulting sequences with those in GenBank. We performed complete blood counts using CELL-DYN 3700 Hematology Analyzer (Abbott, https://www.abbott.com), biochemical profile using a KPM Analytics SAT 450 random access analyzer (https://www.kpmanalytics.com), and protein electrophoresis analyses using Sebia Hydrasys 2 Scan Focusing (, https://www.sebia.com). We calculated 95% CIs for proportions and χ^2^ and odds ratios (OR) to assess differences in prevalence and infection risk stratified by age and sex. We used *t*-tests to compare mean laboratory values between baboons positive and negative for vector-borne pathogens. We considered p values <0.05 statistically significant. 

**Table:** PCR protocols used in study of vector-borne pathogens among baboons, Italy, 2020

Pathogen	Target gene	Primer	Sequence, 5′ →3′	Fragment length, bp	Reference
*Babesia*/*Theileria *spp.	18S rRNA	RLB-F	GAGGTAGTGACAAGAAATAACAATA	460–520	(*2*)
		RLB-R	TCTTCGATCCCCTAACTTTC		
*Ehrlichia/Anaplasma* spp.	16S rRNA	EHR-16SD	GGTACCYACAGAAGAAGTCC	345	(*2*)
		HER-16SR	TAGCACTCATCGTTTACAGC		
*Rickettsia* spp.	*glt*A	CS-78F	GCAAGTATCGGTGAGGATGTAAT	401	(*2*)
		CS-323R	GCTTCCTTAAAATTCAATAAATCAGGAT		
Spotted fever group Rickettsiae	*omp*A	Rr190.70F	ATGGCGAATATTTCTCCAAAA	632	(*2*)
		Rr190.701R	GTTCCGTTAATGGCAGCATCT		
	*omp*B	120–2788	AAACAATAATCAAGGTACTGT	600	(*3*)
		120–3599	TACTTCCGGTTACAGCAAAGT		
*Leishmania infantum*	kDNA minicircle	Leish-1	AACTTTTCTGGTCCTCCG GGTAG	120	(*4*)
		Leish-2	ACCCCCAGTTTCCCGCC		

We included 33 baboons: 21 male, 12 female; 13 juvenile, 16 adult, and 4 elderly. Blood samples from 3/33 (9.1%, 95% CI 3.1%–23.4%; 1 adult male, 1 adult female, 1 juvenile male) were positive for *R. conorii* subsp. *israelensis* by the *glt*A gene; all samples were negative by *omp*A and *omp*B genes. The only sequence type we identified showed 99%–100% nucleotide identity with *R. conorii* subsp. *israelensis* from GenBank; we deposited our sequence in GenBank (accession no. OQ360110). All baboons tested negative for other vector-borne pathogens. 

Although we found adult and male baboons at higher risk for infection (OR 2.6), we found no significant difference by age or sex (p = 0.439). No baboon showed ectoparasitic infestation or clinical signs of vector-borne diseases, and all displayed good physical status (mean complete blood count 3, average bodyweight 17.5 kg). Hematologic and serum chemistry values were within normal ranges (Appendix Tables 1, 2) for both *R. conorii*–negative and –positive baboons (p >0.05). 

Our study revealed a nonnegligible prevalence (9.1%, 3/33) of *R. conorii* subsp. *israelensis* in *P. hamadryas* baboons, representing a pathogen–host association previously demonstrated only among asymptomatic dogs and cats from Portugal (*5*) and in severe cases among symptomatic humans from Italy (*6*). This survey confirms circulation of rickettsiae among baboons, also reported in 1 study of *R. africae* in *P. cynocephalus* yellow baboons from Zambia (*1*). 

Despite routine treatment of baboons (orally administering 0.4 mg/kg ivermectin every 15 days by ground bait), presence of ticks in the zoopark was supported by a previous finding of tickborne pathogens (*A. phagocytophilum*, *Coxiella burnetii*, and *Rickettsia* spp.) in a lion (*7*). Given the baboon grooming behavior of removing ectoparasites from their bodies, lack of *Rhipicephalus* *sanguineus* sensu lato ticks, a vector of rickettsiae (*8*), was not surprising (*9*). However, association between zoopark-dwelling baboons and *Rhipicephalus* spp. ticks, including *R. sanguineus* s.l., is well known (*9*). Because this tick species is prevalent in the study area in all developmental stages, exposure very likely occurs (*10*). 

Taken together, the high density of *P. hamadryas* baboons, their close proximity to the zoopark, and the anthropophilic behavior of *R. sanguineus* s.l. ticks (*10*) highlight the threat to park visitors and workers from *R. conorii* subsp. *israelensis* infection. Absence of clinical signs in positive baboons and lack of differences in hematological and biochemical parameters between negative and positive animals indicate the asymptomatic features of infection and make clarifying the baboons’ role as a potential reservoir more urgent. Measures to control tick ​​circulation should be established to reduce risk for transmission of *R. conorii* subsp. *israelensis* to zoopark visitors and workers. 

Appendix Additional information on study of vector-borne pathogens among baboons in a zoopark in Italy.
