# Supplementary material for: Rickettsia conorii Subspecies israelensis in Captive Baboons
Source: Emerg Infect Dis. 2023 Apr;29(4):841–3. doi: 10.3201/eid2904.221176 (PMC10045702; doi:10.3201/eid2904.221176)
Supplement: Appendix — Additional information on study of vector-borne pathogens among baboons in a zoopark in Italy. [file 22-1176-Techapp-s1.pdf]

Article DOI: https://doi.org/10.3201/eid2904.221176

EID cannot ensure accessibility for supplementary materials supplied by authors. Readers who have difficulty accessing supplementary content should contact the authors for assistance.

Rickettsia conorii subspecies *israelensis* in Captive Baboons

Appendix

Appendix Table 1. Complete blood count and biochemical analyses results from baboons testing negative (n = 30) and positive (n = 3) *to Rickettsia conorii* subspecies *israelensis* DNA, during 2020, in Italy*

Parameter	Units	Mean BabR– †	Mean BabR+ ‡	Standard deviation	Standard error	*t*-test value	*p*-value	Range
Complete blood count	g/dL	12.7	12.2	1.7	1.0	0.5	0.632	12.6 ± 0.9
Hgb	K/µL	442.8	438.7	67.9	41.1	0.1	0.921	316 ± 83
Plt	Femtoliters	7.3	7.9	0.7	0.4	1.4	0.167	8.3 ± 1.0
MPV	K/µL	11.7	9.4	3.3	2.0	1.1	0.314	9.6 ± 2.9
WBC	M/µL	5.2	4.8	0.46	0.3	1.5	0.133	4.95 ± 0.32
RBC	%	40.7	36.6	3.6	2.2	1.8	0.069	38.2 ± 2.5
Hct	Femtoliters	77.6	78.6	2.6	1.6	0.6	0.530	77 ± 2.9
MCV	pg/dL	21.7	22.3	0.9	0.5	1.1	0.279	25.3 ± 0.9
MCH	g/dL	32.5	33.5	1.8	1.1	0.9	0.366	32.9 ± 0.7
MCHC	g/dL	31.7	32.2	1.9	1.1	0.5	0.650	NA
CHCM	pg/dL	3.7	3.4	0.4	0.2	1.2	0.224	NA
CHDW	%	14.0	14.5	0.7	0.4	1.2	0.247	NA
RDW	%	2.1	1.9	0.2	0.1	1.6	0.108	NA
HDW	K/µL	9.1	7.8	3.2	1.9	0.7	0.501	3.3 ± 1.9
Neu	K/µL	1.8	1.2	0.6	0.4	1.6	0.108	2.1 ± 1.3
Lym	K/µL	0.6	0.4	0.2	0.1	1.6	0.108	2.0 ± 2.0
Mon	K/µL	0.03	0.02	0.1	0.06	0.2	0.858	1.0 ± 1.0
Eos	K/µL	0.025	0.03	0.02	0.01	0.2	0.869	0.05 ± 0.05
Bas	%	0.3	0.3	0.07	0.04	0	1.000	NA
‡Biochemical analyses	%	41.2	40.8	3.2	1.9	0.2	0.838	NA
Pct	g/dL	23.1	20.9	3.3	1.9	1.1	0.279	NA
PDW	IU/L	564.0	477.0	622.6	377.0	0.2	0.819	NA
MPC	IU/L	49.6	31.7	16.3	9.8	1.8	0.079	NA
CPK	IU/L	33.4	18.7	32.6	19.7	0.7	0.462	NA
AST	IU/L	642.2	886.0	489.4	296.3	0.8	0.417	NA
ALT	IU/L	22.7	32.7	12.6	7.6	1.3	0.200	NA
ALP	IU/L	5,225.5	5,456.8	1,829.4	1,107.7	0.2	0.836	NA
GGT	mg/dL	0.4	0.3	0.1	0.06	1.6	0.111	NA
Cholinesterase	mEq/L	145.5	142.8	3.5	2.1	1.3	0.212	NA
Total bilirubin	mEq/L	4.2	4.5	0.7	0.4	0.7	0.485	NA
Natrium	mEq/L	22.0	18.7	5.8	3.5	0.9	0.355	NA
Potassium	mEq/L	111.0	107.4	3.5	2.1	1.7	0.099	NA
Natrium/potassium ratio	mmol/L	10.6	13.2	3.7	2.2	1.1	0.255	NA
Chlorine	mg/dL	120.3	105.6	30.5	18.5	0.8	0.432	NA
Anion gap	mg/dL	1.0	0.9	0.3	0.1	0.5	0.587	NA
Glucose	mg/dL	28.1	32.1	10.5	6.3	0.6	0.534	NA
Creatinine	mg/dL	9.0	9.5	0.7	0.4	1.2	0.247	NA
Urea	mg/dL	4.3	3.5	1.5	0.9	0.8	0.385	NA
Calcium	g/dL	6.6	6.8	0.5	0.3	0.7	0.514	NA
Phosphorus	mg/dL	0.18	0.2	0.05	0.03	0.7	0.510	NA
Total proteins	mg/dL	91.7	100.7	18.9	11.4	0.8	0.438	NA
Albumin	mg/dL	50.9	53.4	21.4	12.9	0.1	0.848	NA
Cholesterol	µg/dL	34.7	31.8	5.8	3.5	0.8	0.415	NA
Triglycerides	mmol/L	27.2	25.3	3.2	1.9	0.9	0.334	NA

*BabR–*R. conorii* subsp. *israelensis* DNA–negative baboons, BabR+ *R. conorii* subsp. *israelensis* DNA–positive baboons, HGB (hemoglobin), PLT (platelet), MPV (mean platelet volume), WBC (white blood cell), RBC (red blood cell), Hct (hematocrit), MCV (mean corpuscular volume), MCH (mean corpuscular hemoglobin), MCHC (mean corpuscular hemoglobin concentration), CHCM (cellular hemoglobin concentration mean), CHDW (cell hemoglobin distribution width), RDW (red cell distribution width), HDW (hemoglobin distribution width), Neu (neutrophils), Lym (lymphocytes), Mon (monocytes) Eos (eosinophils), Bas (basophils), Pct (procalcitonin), PDW (platelet distribution width), MPC (mean platelet component), CPK (creatine phosphokinase), AST (aspartate aminotransferase), ALT (alanine aminotransferase), ALP (alkaline phosphatase), GGT (gamma glutamyl transpeptidase).
†BabR–, Baboons tested negative to *Rickettsia conorii* subspecies *israelensis* DNA.
‡BabR+, Baboons tested positive to *Rickettsia conorii* subspecies *israelensis* DNA.

Appendix Table 2. Serum protein electrophoresis in baboons tested negative (n = 33) and positive to *Rickettsia conorii* subspecies *israelensis* DNA, during 2020, in Italy. The protein concentration values are expressed in g/dl

Parameter	Mean value *BabR$^-$	Mean value †BabR$^+$	Standard deviation	Standard error	*t*-test value	*p*-value
Albumin	3.9	4.2	0.6	0.4	0.8	0.415
α-1 globulins	0.2	0.14	0.06	0.04	1.7	0.106
α-2 globulins	0.7	0.5	0.2	0.1	1.6	0.108
β-1 globulins	0.5	0.4	0.1	0.06	1.6	0.111
β-2 globulins	0.5	0.48	0.1	0.06	0.3	0.745
γ-globulins	0.9	0.8	0.3	0.06	1.6	0.111
Total proteins	6.6	6.5	0.5	0.3	0.3	0.744
Albumin/globulins ratio	1.5	1.9	0.5	0.3	1.3	0.196

*BabR–*R. conorii* subsp. *israelensis* DNA–negative baboons, BabR+ *R. conorii* subsp. *israelensis* DNA–positive baboons
